# Pneumocephalus and Facial Droop on an Airplane: A Case Report

**DOI:** 10.5811/cpcem.2020.4.46799

**Published:** 2020-06-10

**Authors:** Irina Sanjeevan-Cabeza, Morgan Oakland

**Affiliations:** Thomas Jefferson University Hospital, Department of Emergency Medicine, Philadelphia, Pennsylvania

**Keywords:** Pneumocephalus, facial droop, Barotrauma

## Abstract

**Introduction:**

Pneumocephalus (PNC) is most commonly associated with trauma or intracranial surgery, less commonly secondary to an infectious source, and is rarely caused by barotrauma.

**Case report:**

A 32-year-old woman presented to the emergency department with complaint of resolved left-sided facial droop and a lingering paresthesia of her left upper extremity after a cross-country flight. Computed tomography demonstrated several foci of air in the subdural space consistent with PNC.

**Conclusion:**

For PNC to occur there must be a persistent negative intracranial pressure gradient, with or without an extracranial pressure change. In this case the pressure change occurred due to cabin pressure.

## INTRODUCTION

Pneumocephalus (PNC) is most commonly associated with trauma or intracranial surgery, less commonly secondary to an infectious source, and is rarely caused by barotrauma.^1^,^2^ We report a case of spontaneous atraumatic PNC in a previously healthy patient who presented with transient facial droop that had completely resolved by the time of presentation. Symptom onset was during a cross-country flight, making barotrauma from cabin pressure changes the suspected etiology.

## CASE REPORT

A 32-year-old woman presented to the emergency department (ED) with complaint of resolved left-sided facial droop and a lingering paresthesia of her left upper extremity. Her medical history was relevant for recurrent otitis media infections; she was otherwise healthy and worked full time. She was not a frequent air traveller, nor did she have a history of scuba diving. Her symptoms began approximately six hours prior to arrival to the ED while she was aboard a flight across the country. She was not coughing, sneezing, or deliberately attempting a Valsalva maneuver when her symptoms started, but as the plane took off she experienced sudden, severe left ear pain and felt left-sided facial as well as left upper extremity numbness. She also felt that her face was “drooping” and when she checked her reflection, she noticed that she had droop on the entire left side of her face: she could not lift her eyebrow, could not smile or frown, was unable to close her eye and was drooling out of the left side of her mouth. She was given a warm compress for her ear by airplane staff and the symptoms resolved within approximately 30 minutes, although her ear pain remained.

Neither her droop nor her numbness was present by the time the plane landed. Her only lingering complaint was that of a “strange sensation” she could not describe in her left upper extremity. She specifically denied sensations of numbness, weakness or paresthesias after the event. Strength and sensation were fully intact. She presented to the ED with these complaints. On exam in the ED, her initial vital signs were within normal limits, and her neurologic exam was completely normal. Her National Institutes of Health Stroke Scale was zero. Her tympanic membranes were intact bilaterally, with subtle bulging of the left concerning for otitis media without signs of rupture.

**Image 1:**
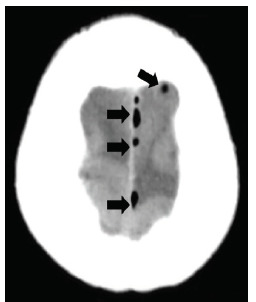
Transverse section of a computed tomography brain where foci of air (black arrows) can be seen along the left aspect of the falx cerebri outside of the superior sagittal sinus.

**Image 2:**
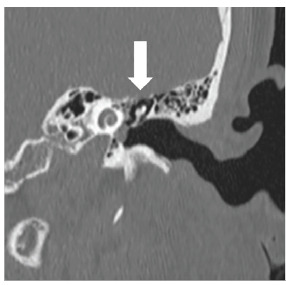
Left coronal section of a computed tomography internal auditory canals demonstrating the patient’s thinned tegmen tympani (white arrow).

Routine laboratory data was unremarkable; however, computed tomography (CT) was notable for small foci of air in the subdural space scattered along the left aspect of the outside of the superior sagittal sinus (Image 1), as well as a focus along the left cerebellar tentorium. There was no midline shift or mass effect. Also noted was pneumatization of the squamosal portion of both temporal bones, and both petrous apices. A CT internal auditory canals was performed (Image 2). Neurology, neurosurgery, and otolaryngology were consulted for management.

Her transient facial droop was attributed to an air pocket near the facial nerve that would have expanded with cabin pressure change, but had since been reabsorbed and therefore was not captured on imaging at the time of the patient’s presentation in the ED. A presumed defect in the dura was discussed by both neurosurgery and otolaryngology, although this defect was not identified on imaging. Otolaryngology recommended placement of a myringotomy tube after discharge from the ED. No acute surgical interventions were indicated as per neurosurgery.

The patient followed up with otolaryngology the following day and had uncomplicated placement of a myringotomy tube with aspiration of a thick mucoid effusion. She reported immediate resolution of her ear pain following placement. She was scheduled to fly back to her hometown and follow up with her local otolaryngology physician, as well as obtain repeat head imaging to confirm resolution of the PNC.

**CPC-EM Capsule**

What do we already know about this clinical entity?

Atraumatic pneumocephalus (PNC) is associated with surgery or infection. It occurs through a fistula that requires a negative pressure gradient between the extracranial and intracranial spaces.

What makes this presentation of disease reportable?

The mechanism (barotrauma) by which this PNC occurred is extremely rare.

What is the major learning point?

Keep PNC in the differential for patients who present with neurological complaints after air travel.

How might this improve emergency medicine practice?

This case highlights the importance of obtaining a thorough travel history and keeping a broad differential diagnosis for patients with recent travel.

## DISCUSSION

This is the first reported case of PNC and facial palsy from altitude barotrauma in emergency medicine (EM) literature. PNC is a rare condition that most commonly occurs as a consequence of trauma or surgical intervention.^1^,^2^ PNC after air travel has been described in isolated case reports where, again, patients had a history of neurosurgical intervention.^3^,^4^ Barotrauma as the etiology of spontaneous PNC is very rare and has been described in scuba diving-related pressure changes.^5^ These cases were identified through a literature search using the keywords “pneumocephalus,” “pneumocranium”, “pneumatocephalus,” or “intracranial pneumatocele” in PubMed and Cochrane Library databases, and are all published in neurosurgical literature, as were the vast majority of PNC case reports identified in our search. Thus, a case of spontaneous PNC after air travel in an otherwise healthy young patient is rare and has not been described in EM literature before.

For PNC to occur there must be a persistent negative intracranial pressure gradient, with or without an extracranial source of positive pressure.^6^

There are two presumed mechanisms as described in neurosurgical literature.^7^ The first mechanism occurs in the setting of low intracranial pressure due to a dural leak or ventricular shunt. In this setting, cerebrospinal fluid is replaced by air. If a fistula exists across the dura to an aerated sinus, air may enter the intracranial space in response to that negative pressure gradient. Postoperative and postprocedure PNC most commonly occurs via this mechanism.

The second mechanism involves “air trapping” in a presumed ball-valve system where two pathologies must be present: a defect in the temporal bone that communicates air from the mastoid cells to the intracranial compartment and a gradient of pressure between the middle ear and the intracranial space. Pressure changes can be both internal (such as Valsalva) and external (ambient pressure). Once the air is inside the intracranial space it increases intracranial pressure again, collapsing or obliterating the fistula after equalization of pressures and trapping it inside as the negative gradient is terminated.

This patient demonstrates the second mechanism described, as the cabin pressure change and presumed temporal bony defect were both present. Cabin pressure decreases rapidly during ascent, causing gases to expand. The air in the patient’s left middle ear expanded in a fixed space, likely finding the path of least resistance internally, via the temporal bone defect and subsequent dural defect, instead of through the thick mucoid effusion and her tympanic membrane, externally. It was then trapped intracranially after the pressure gradient equalized.

The patient’s facial droop was left-sided and peripheral (involved upper and lower facial palsy) as per her description; therefore, this was likely caused by an air pocket near the facial nerve that would have expanded with cabin pressure change, causing ipsilateral symptoms. It is unclear why this resolved with a warm compress; however, heat lends gas particles kinetic energy and so the warmth could have caused the air pocket to dissipate. Her improvement with placement of the myringotomy tube one day later also correlates with this explanation.

## CONCLUSION

Given the episodic nature of the facial palsy, the air pocket near the facial nerve was suggested as a likely etiology, but was not captured on imaging since that symptom resolved with the presumed resorption of the air. Unfortunately we were unable to obtain records from the patient’s follow-up appointments, nor could we obtain follow-up imaging. This case report highlights the importance of keeping barotrauma in the differential diagnosis for patients who present with neurological and otologic complaints after air travel.
